# Clinical Application of Indocyanine Green Fluorescence Imaging in the Resection of Hepatoblastoma: A Single Institution's Experiences

**DOI:** 10.3389/fsurg.2022.932721

**Published:** 2022-06-30

**Authors:** Yuanchao Shen, Manna Zheng, Jiahao Li, Tianbao Tan, Jiliang Yang, Jing Pan, Chao Hu, Yan Zou, Tianyou Yang

**Affiliations:** Department of Pediatric Surgery, Guangzhou Women and Children's Medical Center, Guangzhou Medical University, Guangzhou, China

**Keywords:** indocyanine green, fluorescence imaging, hepatoblastoma, children, hepatectomy

## Abstract

**Purpose:**

Indocyanine green (ICG) fluorescence imaging is becoming increasingly popular in adult oncologic surgery, but remains relatively uncommon in pediatric oncologic surgery. Herein, we report our experience with the use of ICG fluorescence imaging in the resection of hepatoblastoma (HB).

**Patients and Methods:**

Hepatoblastoma patients who underwent liver resection with ICG fluorescence imaging between January 2020 and March 2021 were included in this study. Patients’ demographic data, clinical information, and detailed information of the use of ICG fluorescence imaging were retrospectively reviewed.

**Results:**

Sixteen HB patients underwent ICG fluorescence imaging-guided liver resection. There were 11 males and 5 females, age ranged from 8 to 134 months. The initial alpha-fetoprotein ranged from 436 to 528,390 ng/ml. There were one pre-treatment extent of tumor stage I, nine stage II, four stage III, and two stage IV. Three patients underwent up-front hepatectomy, 13 patients received 2–8 cycles of platinum-based neoadjuvant chemotherapy and underwent delayed hepatectomy. ICG (0.5 mg/kg) was given intravenously 48–72 h prior to surgery. The operative time ranged from 180 to 400 min. All patients achieved negative surgical margins. In two patients, ICG identify additional lesions which were not detected in preoperative imaging.

**Conclusion:**

ICG fluorescence imaging is useful in the resection of HB and may detect small lesions not shown in preoperative imaging.

## Introduction

Hepatoblastoma (HB) is the most common primary pediatric liver malignancy, which accounts for approximately 1% of all pediatric malignancies. The estimated incidence of HB is about 1–1.5 cases per million per year in children younger than 15 years (1–3). The management of HB is multi-disciplinary, consisting of chemotherapy and surgery. Complete surgical resection is key to successful treatment of HB (4, 5). During the past several decades, major advances had been achieved in the surgical management of HB. Extended liver resection for pre-treatment extent (PRETEXT) III and PRETEXT IV patients has been investigated, with encouraging results. Liver transplantation for advanced HB is gaining more acceptance, with a 5-year overall survival rate of over 70% (6).

In recent years, indocyanine green (ICG) fluorescence imaging-guided liver resection has become increasingly popular (7, 8). Several studies have described the use of ICG in the resection of HB, with successful results. After intravenously injection, ICG is secreted into bile and washed out in hours within normal liver, whereas ICG secretion is inhibited in malignant liver tumor and metastasis focus. Based on this characteristic, ICG fluorescence imaging navigation becomes an effective technology in hepatobiliary surgery. It can be used to determine tumor margin, detect satellite lesions, and other many applications. Hereby, we summarize our experience with the use of ICG fluorescence imaging-guided HB resection.

## Patients and Methods

HB patients who underwent hepatectomy with the use of ICG fluorescence imaging between January 2020 and March 2021 were included in this study. All patients underwent computed tomography (CT) scanning, and a PRETEXT stage was assigned. Patients underwent either up-front hepatectomy or ultrasound-guided percutaneous biopsy with neoadjuvant chemotherapy based on clinical findings, especially the PRETEXT stage. The diagnosis of HB was established based on pathologic findings. Patients’ demographic, disease characteristics, surgical finding and ICG fluorescence imaging, pathologic diagnoses, and clinical outcome were reviewed and analyzed.

This study was approved by the Guangzhou Woman and Children's Medical Center's Institutional Review Board. All patients’ data were anonymized and deidentified prior to analyses. ICG (0.5 mg/kg) was given intravenously 48–72 h prior to surgery, which was in accordant with the recommendations in consensus guidelines for the use of fluorescence imaging in hepatobiliary surgery (9). Informed consent was obtained from the patients’ parents before ICG injection.

**Figure 1:**
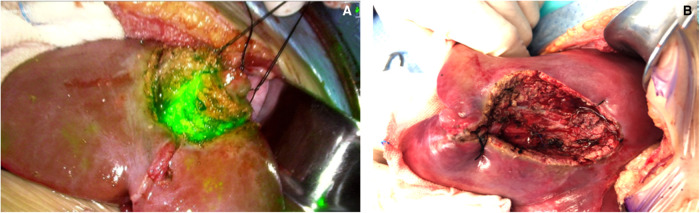
Real-time modified resection plane by indocyanine green (ICG) fluorescence imaging (**A**) and after complete resection (**B**).

During the surgery, a near-infrared light camera was used to detect ICG fluorescence. Resection lines can be marked based on real-time ICG fluorescence imaging (Figure 1), and small satellite lesions can also detected. During and after the completion of liver resection, the resection plane and surgical margin were re-examined with ICG fluorescence imaging.

### Statistical Analyses

The statistical analyses were performed using SAS software, version 9.4 (SAS Institute, Inc., Cary, NC, USA). The continuous variables are presented as the medians (ranges), while the categorical variables are presented as numbers (percentages).

## Results

Sixteen patients underwent ICG fluorescence imaging-guided hepatectomy (Table 1). There were 11 (68.8%) males and 5 (31.2%) females. The median age was 15 months (8–134 months). There were 1 (6.2%) cases of PRETEXT stage I, 9 (56.2%) cases of stage II, 4 (25.0%) cases of stage III, and 2 (12.5%) cases of stage IV. The median tumor diameter based on the preoperative CT images was 8 cm (2.9–11.0 cm). The median preoperative alpha-fetoprotein (AFP) value was 97,983.5 ng/ml (436–528,390 ng/ml). Three (18.7%) patients underwent up-front hepatectomy, and 13 (81.2%) received 2–8 cycles of platinum-based neoadjuvant chemotherapy prior to hepatectomy.

**Table 1:** Patients’ demographic and clinical information.

	Gender	Age (month)	PRETEXT	Neoadjuvant chemotherapy (cycles)	POSTTEXT	Tumor location	Tumor size (cm)	Operative time (minutes)	Volume of red blood cells transfusion (u)	Pathologic subtype
1	M	9	II	3	III	S5, S6	6.5 × 6.4 × 8.5	265	1	EM
2	M	8	II	6	II	S5, S8	2.9 × 2.8 × 2.5	300	1	MEM
3	F	12	II	2	II	S3, S4	4.6 × 3.6 × 4.3	235	0.5	MEM
4	M	48	II	5	II	S5, S6, S7	8.6 × 7.2 × 11	400	2	MEM
5	M	12	II	0	–	S5, S6	8.1 × 5.9 × 7.8	180	0	EM
6	F	29	III	2	II	S4, S5, S6	–	240	1	EV
7	M	32	II	2	II	S7, S8	6.9 × 5.9 × 8.4	295	0.8	MEM
8	M	16	II	2	II	S4	3.1 × 2.9 × 2.0	260	0.2	EV
9	M	15	III	6	III	S4, S6, S7, S8	6.8 × 5.9 × 9.3	360	1	MEM
10	M	11	I	0	–	S5	4.9 × 4.6 × 4.1	192	0.5	EM
11	M	19	II	2	II	S5, S6, S7, S8	7.1 × 5.1 × 9.3	340	0.8	EM
12	M	23	IV	7	IV	S2, S3, S4, S5, S6, S8	7.3 × 4.0 × 4.9	265	1.5	EV
13	F	39	II	0	–	S5, S6	10.1 × 6.3 × 10.6	120	0	EV
14	F	14	III	2	III	S2, S3, S4, S7	4.6 × 3.9 × 2.7	205	1	EV
15	M	132	IV	8	II	S5, S6, S7, S8	8.0 × 4.1 × 4.8	279	1	MEM
16	F	14	III	7	II	S4, S5, S7, S8	4.2 × 2.7 × 3.0	270	1	EV

*PRETEXT*, *pre-treatment extent of disease system; POSTTEXT*, *post-treatment extent of disease system; M*, *male; F*, *female; EM, epithelial mixed; MEM, Mixed epithelial and mesenchymal; EV, Epithelial variants.*

**Figure 2:**
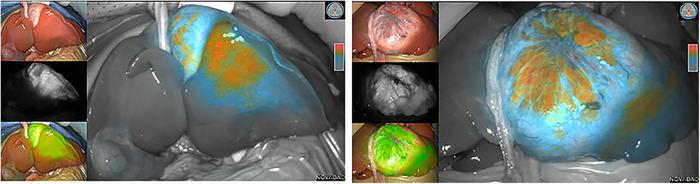
Intraoperative (ICG) fluorescence imaging shows remarkable contrast between tumor (fluorescence color) and normal liver parenchymal.

**Figure 3:**
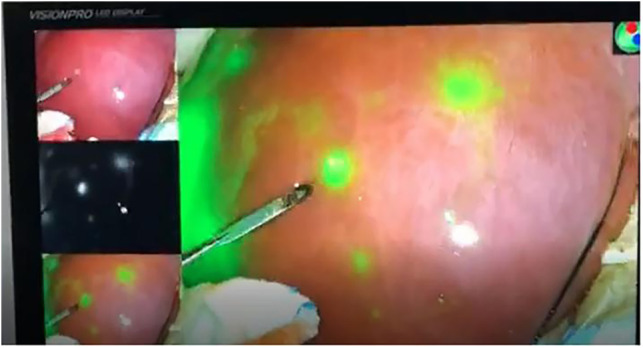
Multiple small lesions were detected by (ICG) fluorescence.

**Figure 4:**
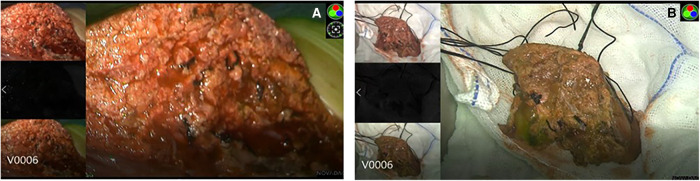
No fluorescence was detected at the liver resection plane *in situ* (**A**) and *ex vivo* (**B**).

During surgery, the boundary between normal liver tissue and HB was presented under the real-time ICG fluorescence imaging in 16 patients (Figure 2). Multiple small lesions were detected by intraoperative ICG fluorescence imaging in two patients (patient 1 and patient 3, Table 1). These lesions were not found in preoperative contrast-enhanced CT with iopromide and contrast-enhanced MRI with Gd-DTPA-BMA, and were confirmed as HB tissue pathologically (Figure 3). ICG fluorescence imaging was used to re-examine the resection plane *in situ* and *ex vivo*, none of them show fluorescence (Figure 4). This was consistent with the pathologic findings of negative surgical margins in 16 patients.

The median operation time was 265 min (80–400 min). All patients achieved negative surgical margins. There were 6 (37.5%) cases of epithelial variants type, 6 (37.5%) cases of mixed epithelial and mesenchymal type, and 4 (25.0%) cases of epithelial mixed type. All patients had an uneventful recovery.

## Discussion

Our study showed that intraoperative ICG fluorescence imaging could be a valuable tool for HB surgery. It helps to determine the resection plane and assure a negative resection margin. Furthermore, it can detect small lesions not shown in preoperative imaging. Our results were consistent with several other studies, showing that ICG aids in determining the resection line and identifying residual tumors (10–12).

ICG has been used in clinic for more than 50 years, and the overall adverse effect rate is less than 0.01% (13). ICG is relatively safe, the lethal doses of ICG is up to 50–80 mg/kg, it is essentially nontoxic when injected at standard doses of less than 2 mg/kg (14). No patients have any adverse effect after ICG injection in our cohort.

ICG fluorescence imaging is widely used in surgical navigation, but its clinical application in pediatric surgery is still in its infancy (15). The use of preoperatively injected intravenous ICG and near-infrared imaging equipment has emerged as a novel therapy in cases of HB and metastatic resections (16–18). As is well known, ICG is retained in the tumor tissue much longer than in the normal liver parenchyma (19). By this principle, ICG can be used for imaging both primary HB and metastases. Using this specialized equipment, sites of primary HB and metastatic locations are able to be detected with green fluorescence (4). This modality can be used to help achieve a negative resection status, safely remove tumors from closely underlying vascular structures, as well as assess the degree of any remaining malignancy (4). The negative margin can be achieved and confirmed through repeatedly imaging the resection plane during hepatectomy, and imaging the resection margin after tumor removal. All 16 patients in our study achieved negative resection margin. In addition to delineate the resection line and assess the degree of any remaining malignancy. The identification of small lesions that were not detected in preoperative CT/MRI scanning under ICG fluorescence imaging was observed in our study, showing that the significant advantage of ICG fluorescence imaging is compared with preoperative imaging examination (20). We identified multiple small lesions in two cases with this method. ICG fluorescence imaging is also useful in identifying small viable metastatic lung lesions. Kitagawa et al. reported that ICG can detect lung lesions as small as 0.062 mm in diameter, and all of the pathologically positive lesions were clearly fluorescence positive in a study of 10 patients (21).

However, ICG fluorescence imaging has some limitations. One of the limitations of ICG fluorescence imaging is its inability to probe deep tissue. The fluorescence emitted by ICG can only penetrate 5–10 mm of tissue. When the tumor goes beyond this depth, its fluorescence is undetectable on the surface (22, 23). Souzaki et al. previously reported that ICG failed to detect a tumor 12 mm from the lung surface (10). Additionally, although ICG is highly sensitive to tumors, its specificity is rather low. Cotoh's study reported that strong fluorescence also can be displayed in the surface of focal cirrhosis and hepatic nodular hyperplasia, the false positive rate of these lesions can reach 40%–50% (24). Dysfunction liver can present fluorescence as well. Some non-tumor tissues showing fluorescence are hard to avoid. When intraoperative ICG fluorescence imaging was significantly different from the preoperative imaging results, the possibility of false positive must be considered, and intraoperative frozen-section can be helpful in determining the nature of suspicious nodule. Further study is needed to find out how to reduce the false positive rate such as by developing high-performance near-infrared nanocomplexes formulated with ICG with high selectivity for tumors (25). This is a small case-series report, and further studies with a larger number of patients will be needed to validate the ICG fluorescence imaging technique.

## Conclusion

In conclusion, ICG fluorescence imaging is useful in the resection of HB. This technique effectively improves the surgical efficiency and helps achieve complete resection of tumors.

## Data Availability

The original contributions presented in the study are included in the article/Supplementary Material, further inquiries can be directed to the corresponding author/s.

## References

[1] Sharma D Subbarao G Saxena R. Hepatoblastoma. Semin Diagn Pathol. (2017) 34(2):192–200. 10.1053/j.semdp.2016.12.015 28126357

[2] Aronson DC Meyers RL. Malignant tumors of the liver in children. Semin Pediatr Surg. (2016) 25(5):265–75. 10.1053/j.sempedsurg.2016.09.002 27955729

[3] Musick SR Smith M Rouster AS Babiker HM. Hepatoblastoma. In: *StatPearls* [Internet]. Treasure Island, FL: StatPearls Publishing (2022). PMID: 30521216

[4] Yang T Whitlock RS Vasudevan SA. Surgical management of hepatoblastoma and recent advances. Cancers (Basel). (2019) 11(12):1944. 10.3390/cancers11121944 PMC6966548 31817219

[5] Malogolowkin MH Katzenstein HM Meyers RL Krailo MD Rowland JM Haas J Complete surgical resection is curative for children with hepatoblastoma with pure fetal histology: a report from the children’s oncology group. J Clin Oncol. (2011) 29:3301–6. 10.1200/jco.2010.29.3837 21768450 PMC3158601

[6] Trobaugh-Lotrario AD Meyers RL Tiao GM Feusner JH. Pediatric liver transplantation for hepatoblastoma. Transl Gastroenterol Hepatol. (2016) 1:44. 10.21037/tgh.2016.04.01 28138611 PMC5244811

[7] Nomi T Hokuto D Yoshikawa T Matsuo Y Sho M. A novel navigation for laparoscopic anatomic liver resection using indocyanine green fluorescence. Ann Surg Oncol. (2018) 25(13):3982. 10.1245/s10434-018-6768-z 30218249

[8] Nishino H Hatano E Seo S Nitta T Saito T Nakamura M Real-time navigation for liver surgery using projection mapping with indocyanine green fluorescence: development of the novel medical imaging projection system. Ann Surg. (2018) 267(6):1134–40. 10.1097/SLA.0000000000002172 28181939

[9] Wang X Teh CSC Ishizawa T Aoki T Cavallucci D Lee SY Consensus guidelines for the use of fluorescence imaging in hepatobiliary surgery. Ann Surg. (2021) 274(1):97–106. 10.1097/SLA.0000000000004718 33351457

[10] Souzaki R Kawakubo N Matsuura T Yoshimaru K Koga Y Takemoto J Navigation surgery using indocyanine green fluorescent imaging for hepatoblastoma patients. Pediatr Surg Int. (2019) 35(5):551–7. 10.1007/s00383-019-04458-5 30778701

[11] Esposito C Del Conte F Cerulo M Gargiulo F Izzo S Esposito G Clinical application and technical standardization of indocyanine green (ICG) fluorescence imaging in pediatric minimally invasive surgery. Pediatr Surg Int. (2019) 35(10):1043–50. 10.1007/s00383-019-04519-9 31273452

[12] Abdelhafeez A Talbot L Murphy AJ Davidoff AM. Indocyanine green-guided pediatric tumor resection: approach, utility, and challenges. Front Pediatr. (2021) 9:689612. 10.3389/fped.2021.689612 34616696 PMC8489593

[13] Speich R Saesseli B Hoffmann U Neftel KA Reichen J. Anaphylactoid reactions after indocyanine-green administration. Ann Intern Med. (1988) 109(4):345–6. 10.7326/0003-4819-109-4-345_2 3395048

[14] Takahashi H Zaidi N Berber E. An initial report on the intraoperative use of indocyanine green fluorescence imaging in the surgical management of liver tumors. J Surg Oncol. (2016) 114(5):625–9. 10.1002/jso.24363 27611115

[15] Lau CT Au DM Wong KKY. Application of indocyanine green in pediatric surgery. Pediatr Surg Int. (2019) 35(10):1035–41. 10.1007/s00383-019-04502-4 31243546

[16] Fernández-Bautista B Mata DP Parente A Pérez-Caballero R De Agustín JC. First experience with fluorescence in pediatric laparoscopy. European J Pediatr Surg Rep. (2019) 7(1):e43–6. 10.1055/s-0039-1692191 PMC6611721 31285982

[17] Yanagi Y Yoshimaru K Matsuura T Shibui Y Kohashi K Takahashi Y The outcome of real-time evaluation of biliary flow using near-infrared fluorescence cholangiography with indocyanine green in biliary atresia surgery. J Pediatr Surg. (2019) 54(12):2574–8. 10.1016/j.jpedsurg.2019.08.029 31575415

[18] Rentea RM Halleran DR Ahmad H Sanchez AV Gasior AC McCracken K Preliminary use of indocyanine green fluorescence angiography and value in predicting the vascular supply of tissues needed to perform cloacal, anorectal malformation, and Hirschsprung reconstructions. Eur J Pediatr Surg. (2020) 30(6):505–11. 10.1055/s-0039-1700548 31858494

[19] Tebala GD Bond-Smith G. Indocyanine green fluorescence in elective and emergency laparoscopic cholecystectomy. A visual snapshot. Surg Technol Int. (2020) 37:69–71. PMID: 33031562

[20] Nguyen QT Olson ES Aguilera TA Jiang T Scadeng M Ellies LG Surgery with molecular fluorescence imaging using activatable cell-penetrating peptides decreases residual cancer and improves survival. Proc Natl Acad Sci USA. (2010) 107(9):4317–22. 10.1073/pnas.0910261107 20160097 PMC2840114

[21] Kitagawa N Shinkai M Mochizuki K Usui H Miyagi H Nakamura K Navigation using indocyanine green fluorescence imaging for hepatoblastoma pulmonary metastases surgery. Pediatr Surg Int. (2015) 31(4):407–11. 10.1007/s00383-015-3679-y 25667048

[22] Ishizawa T Tamura S Masuda K Aoki T Hasegawa K Imamura H Intraoperative fluorescent cholangiography using indocyanine green: a biliary road map for safe surgery. J Am Coll Surg. (2009) 208(1):e1–4. 10.1016/j.jamcollsurg.2008.09.024 19228492

[23] Ishizawa T Masuda K Urano Y Kawaguchi Y Satou S Kaneko J Mechanistic background and clinical applications of indocyanine green fluorescence imaging of hepatocellular carcinoma. Ann Surg Oncol. (2014) 21(2):440–8. 10.1245/s10434-013-3360-4 24254203

[24] Gotoh K Yamada T Ishikawa O Takahashi H Eguchi H Yano M A novel image-guided surgery of hepatocellular carcinoma by indocyanine green fluorescence imaging navigation. J Surg Oncol. (2009) 100(1):75–9. 10.1002/jso.21272 19301311

[25] Egloff-Juras C Bezdetnaya L Dolivet G Lassalle HP. NIR fluorescence-guided tumor surgery: new strategies for the use of indocyanine green. Int J Nanomedicine. (2019) 14:7823–38. 10.2147/IJN.S207486 31576126 PMC6768149

